# Reducing the use of seclusion for mental disorder in a prison: implementing a high support unit in a prison using participant action research

**DOI:** 10.1186/1752-4458-6-2

**Published:** 2012-04-09

**Authors:** Yvette Giblin, Andy Kelly, Enda Kelly, Harry G Kennedy, Damian Mohan

**Affiliations:** 1 National Forensic Mental Health Service, Central Mental Hospital, Dundrum, Dublin 14, Ireland; 2 Irish Prison Service, Mountjoy Prison Complex, North Circular Road, Dublin 7, Ireland; 3 Department of Psychiatry, Trinity College, Dublin, Ireland

**Keywords:** Sentenced Prison, Seclusion, Risk stratification

## Abstract

**Background:**

Vulnerable prisoners and mentally disordered offenders who present with risk of harm to self or others were accommodated in Special Observation Cells (SOCs) isolated from others for considerable periods of time. This practice has been criticised by the Council of Europe Committee for the Prevention of Torture. The objective of this initiative was to reduce the use of seclusion within the prison and to improve the care of vulnerable and mentally ill prisoners within the prison.

**Results:**

The prison studied is a committal centre for sentenced prisoners with an official bed capacity of 630. The forensic mental health in-reach team, in co-operation with the prison health service followed the 'spiral' of planning, action and fact finding about the results of the action. In December 2010 a 10 bed High Support Unit (HSU) was established within the prison. During the first year, 96 prisoners were admitted. A third (35%) reported psychotic symptoms, 28% were referred due to the immediate risk of self-harm, 17% were accommodated for medical treatments and increased observation, 13% received specialised treatment by the Addiction Psychiatry team, 6% presented with emotional distress. One prisoner was accommodated on the HSU due to the acute risk he posed to others. A major mental illness was diagnosed in 29%, 20% required short-term increased support for crisis intervention and were found not to have a mental illness. A further 10% were deemed to be feigning symptoms of mental illness to seek refuge in the HSU. 7% had personality disorder as their primary diagnosis and 4% had a learning disability. Stratifying risk within the prison population through the provision of the HSU decreased the total episodes of seclusion in the prison by 59% (*p *< 0.001) in addition to providing a more effective psychiatric in-reach service to the prison. Pathways between the prison and the forensic psychiatric hospital saw no change in activity but improved continuity of care.

**Conclusions:**

The next step is to further stratify risk by establishing a low support unit to serve as a step-down from the high support unit.

## Background

In many jurisdictions prisons do not have a cap on numbers and are prone to overcrowding. Large numbers of mentally ill persons are committed to prisons internationally [1] and this has been confirmed in the prison population described here [2]. International conventions on human rights such as the Universal Declaration of Human Rights [3] and European Convention on Human Rights [4] have set out that prisoners retain all their human rights including the rights to life, freedom from torture, privacy and family life. These rights have been elaborated specifically for prisoners in the UN Standard Minimum Rules for the Treatment of Prisoners [5] and the Council of Europe Committee for the Prevention of Torture (CPT) Standards [6]. These set out that there should be an equivalence of standards of medical and psychiatric care for prisoners and those in the community, and that the "insane" should be treated in hospital, not in prisons. The Irish Prison Service has been visited regularly by the CPT and this project arose in response to these inspection reports [7]

Prisons are unable to carry out pre-admission (pre-committal) assessments of the needs and risks of new in-mates. In practice, prisons assess the risks and needs of new inmates on reception and respond primarily by allocating or stratifying prisoners to locations appropriate to their need. This is more difficult to do in prisons where there are large numbers of new receptions. Further, this process is dominated by the need to manage risks of violence by and between prisoners [6], pp23-25, with physical and mental health needs often subordinated to security and good order in the prison's system of priorities.

### Rationale

This project was driven by two needs. The first was a recognised need to reduce the use of special observation (isolation) cells (SOCs) in the prison. Extra resources were not available for enhanced staffing. A solution was required that could reduce the use of SOCs without any increase in injuries or self harm, and by means of re-organising existing resources. The second need arose from the necessity to provide for those sentenced prisoners with major mental illnesses who had been transferred to a forensic psychiatric hospital where they had responded well to treatment. If returned to prison to serve out the remainder of their sentence however, they were prone to relapse due to ready availability of cannabis and other drugs in prison wings, and the stresses of over-crowding and interactions with other prisoners. If such prisoners could be safely returned to finish their sentences by clarifying a safe pathway, much more efficient use could be made of scarce secure hospital places.

### Objectives

The objective of this project was to establish a systemic form of risk-need responsivity for the mental health needs of prisoners who were not so ill as to require hospitalisation. The first objective of this phase of the project was to reduce the use of special observation (isolation) cells.

## Methods

### Study design

A process of participatory action research best described the approach required. This consists of comparative research on the conditions and effects of various forms of intervention or re-organisation and research leading to social action. This has been described as a spiral of steps each of which consists of planning, action and fact finding about the result of the action [8]. Central to this is the involvement of all stakeholders, including service providers and managers of all agencies involved. The service users - mentally disordered prisoners - had been extensively surveyed some years earlier [2,9]

### Case description: catalysts for change

The need for dealing with both the increased morbidity and mortality in prisons including the prison studied has been highlighted by many different bodies and indeed by the prison inspectorate itself. The present study concentrates however on the need to decrease the use of special observation cells and improve the pathways for mentally disordered prisoners to access care and treatment.

The 1991 Report of the Advisory Group on Prison Deaths [10] amongst its 57 recommendations, highlighted the need for improved psychiatric input to prisons. The Report of the National Steering Committee on Prison Deaths, 1999 [11] reviewed all deaths in prison since 1991 and revisited the recommendations made in the 1991 Report of the Advisory Group on Prison Deaths. There were thirty-two deaths by suicide in custody from 1991 to 1999, eighteen (56%) of which were in the prison studied, highlighting the need for improved management of risk of self harm and suicide within the prison.

Over the past 20 years the CPT has undertaken five preventative inspections of prison health services in Ireland [7] and expressed concerns relating to the welfare of mental health services to prisoners on each occasion. Upon completion of its 2002 visit, the delegation requested that the authorities in the jurisdiction take immediate steps to bring to an end the practice of holding mentally ill patients in padded cells in prison and to ensure their transfer, without delay, to an establishment capable of offering them the treatment required by their condition, such as the Central Mental Hospital or local catchment area psychiatric hospital [12]. The CPT's delegation found that at the prison studied here, prisoners in need of psychiatric care were frequently placed in unfurnished padded cells which had poor lighting and were dirty. They found that the practice observed in this prison was likely to contribute to the deterioration of the mental state of the prisoners concerned and was described as anti-therapeutic and characterised as inhuman and degrading. It was following this report that Special Observation Cells (SOC) were introduced. A Special Observation Cell is defined as a cell "so constructed and designed, and incorporating such exceptional safety features, furnishings and methods of observation, as to afford enhanced safety for the prisoner accommodated therein, including safeguarding against self-harm" [12].

However, on a subsequent visit in 2010 [13], the CPT remained concerned about the use of SOCs in the prison. Concerns were expressed about the over use of SOC cells, including the use of SOC cells for management and punishment. Moreover, the use of an SOC is likely to be in breach of Principle 9 of the United Nations principles regarding the protection of persons with mental illness [14]. Principle 9 states that "Every patient shall have the right to be treated in the least restrictive environment and with the least restrictive or intrusive treatment appropriate to the patient's health needs and the need to protect the physical safety of others."

A national policy for mental health services *Vision for Change *[15] set out a comprehensive policy framework for mental health policy. This endorsed the principle that prison mental health services should be integrated and coordinated with social work, psychology and addiction services to ensure provision of integrated and effective care. The policy did not refer specifically to the needs of violent or vulnerable prisoners.

The prison studied here has attracted media attention due to a number of adverse incidents involving suicides and homicides within the prison. Since 2005 there have been two homicides in custody.

### The need to stratify risk in prison

Stratification of risk in a mental health setting refers to placement of patients in an environment that addresses the risk they present but, in keeping with Principle 9.1 of United Nations principles for mental health, imposing the minimum necessary restrictions and intrusions [14]. Forensic mental health facilities have evolved working practices and policies, so that patients admitted to hospitals are stratified according to the levels of need for care [16]. Accommodating prisoners with increased mental health need in a purposeful environment and segregated from the main prison population improves therapeutic assessment and treatment where necessary, in a safer environment.

Historically, in the prison studied, at-risk prisoners were accommodated in isolation cells or Special Observation Cells (SOCs) for considerable periods of time. Prisoners were either managed in an SOC or on ordinary location. There was an absence of any other facility to manage risk within the prison. It was this hiatus that led to the development of the High Support Unit.

Over a 3 year period an agreement was arrived at between the in-reach forensic mental health team and the Irish Prison Service health managers that of the options considered, a High Support Unit within the prison studied was the most practical and most likely to achieve the goals desired. This plan was widely canvassed amongst stakeholders including service deliverers in the prison as well as senior managers.

### Setting

The prison studied is a closed, medium security prison for adult males. The prison population of the prison studied is made up of individuals from a variety of backgrounds but the general trend is one of serious social and educational disadvantage [2]. It is the main committal prison for sentenced prisoners in Dublin city and county and the oldest penal institution in the State. International studies have long established that the risk of having a serious mental illness is substantially higher in prisoners than in the general population [1]. Prison Morbidity Studies [2,9,17-20] carried out in the prison studied here are consistent in demonstrating an unacceptably high level of psychiatric morbidity relative to the general population.

The prison studied has an operational capacity for 630 prisoners. Overcrowding adds to the difficulty of detaining prisoners in a safe and secure environment. There was a sharp rise from 3010 committals in 2008 to 4128 committals in 2009. In 2010, 4465 prisoners were committed into custody in the prison studied.

### The high support unit (HSU)

The unit consists of a 10-bed High Support Unit. It provides a dedicated area within the prison where mentally ill and vulnerable prisoners, who present with a risk of harm to self or to others, can be separated from the general prison population and closely monitored in a safer environment. Every effort is made to make this a drug-free area within the prison. It is acknowledged that most illicit drugs are available in the ordinary locations (wings) of the prison.

In November 2010, prior to the unit becoming operational, joint training sessions were provided by the National Forensic Mental Health Service staff and Irish Prison Service nursing staff for the prison officers. The prison authorities ring-fenced training time for the officers to attend. Topics included suicide awareness, risk assessment, psychiatric screening and the role of the visiting mental health team. Prison officers expressed initial fears about the risks associated with mental illness and concerns regarding stigma. These were addressed through educational workshops. A need was identified to change custom and practice within the prison. Heretofore, prison staff felt much safer when behaviourally disturbed prisoners who were perceived to be more dangerous were placed in isolation. The challenge was to demonstrate that this group of prisoners could be safely managed by increasing staff supervision within a dedicated area rather than locking them for prolonged periods in isolation, having to wear refractory clothing and with minimal human contact, apart from their food and sanitation needs.

The primary aim of the psychiatric in-reach team at the Health Service National Forensic Mental Health Service and local nurse managers in seeking to set up the HSU was to reduce the frequency and duration of time spent by prisoners in SOC cells, through providing an alternative environment that which was less restrictive and provided increased observation and interaction with staff. There was regular input by staff from the National Forensic Mental Health Service. Liaison included a weekly multi-agency meeting between clinical and prison staff held in the HSU. Each prisoner's treatment plan, progress and future placement was reviewed on a weekly basis.

Psychiatric In-reach services are provided by visiting psychiatrists and a community forensic mental health nurse (CFMHN) from the National Forensic Mental Health Service, a specialist tertiary mental health service funded and managed by the state health service, not by the Department of Justice. In addition to being involved in the day to day assessment and management of patients on the HSU, the CFMHN assesses prisoners in the main prison and provided continuity of nursing care when a prisoner is discharged from the HSU to the general prison population. For those being discharged to the community, the Probation service provides input with post sentence discharge planning.

Table 1 compares access to mental health and other services in the high support unit and compares the regime in an ordinary prison wing.

**Table 1:** access to mental health services on ordinary wings and high support unit compared

	Ordinary prison wing	High support unit
Number of cells	35	10

Number of prison officers per shift	4	3

Attendance of prison health care manager	As required. Based in main prison surgery.	daily

Hours of lock-down (confined to cells)	16.5	16.5

Nurses attend on the wing	When requested	Every day

Community mental health nurses attend on the wing	Clinics in main prison surgery for those with appointments	Three times a week and as requested

Psychiatrists attend on the wing	Twice weekly clinics in main prison surgery for those with appointments (for 630 inmates)	Three times a week and as requested for the ten inmates.

Multi-disciplinary/multi-agency reviews each week	no	yes

The HSU opened in December 2010. Clinical and administrative characteristics of prisoners admitted to the unit over the first year were analysed.

### Participants

Referrals to the High Support Unit are accepted from nurses screening newly sentenced prisoners in the reception area who were thought to either be a risk to themselves or show signs of mental illness, or needed increased nursing observation. Prisoners already allocated to locations within the prison could be referred by nurses, general practitioners or visiting psychiatrists, chaplains, teachers or probation officers often following a request for assessment by discipline staff or governors. Family members and prisoners themselves initiated referrals also. Gate keeping for admissions is maintained by the nurses and healthcare manager. Discharges are decided by a multi-agency group including the visiting psychiatrists from the forensic mental health service, prison nurse managers, probation service and health care governors.

### Variables, data sources and measurements

A register was kept of all incidents of use of the SOCs for medical reasons across the prison for the period of 11 months prior to the opening of the HSU (January 2010 to November 2010) and the first 12 months of operation of the HSU (December 2010 to November 2011). It was not possible to collate the duration of episodes of use of the SOCs prior to the introduction of the HSU.

A register was kept of all admissions to the HSU. A statutory register was maintained at the Central Mental Hospital of all admissions from the prison studied. This is the only designated centre legally empowered to accept such admissions.

Diagnoses were made in accordance with ICD-10 [21].

### Statistical methods

The number of seclusion episodes each month for the 11 months prior to the introduction of the HSU and the number for each month after the introduction of the HSU were compared using SPSS for both analysis of variance and non-parametric tests.

The numbers of transfers from the prison to the forensic psychiatric hospital for 12 months before and after the introduction of the HSU were also compared using SPSS.

## Results

### Case study: descriptive data

#### Admissions

During the first year of operation of the HSU, 96 prisoners were admitted to the HSU facility from the other locations in the prison. A third (35%) of all accommodated on the HSU reported psychotic symptoms. A further 28% were referred due to the immediate risk of self-harm. 17% were accommodated on the unit due to the need for various medical treatments and increased observation. 13% of prisoners were referred for specialised treatment by the Addiction Psychiatry team. 6% of prisoners presented with emotional distress in the context of stressors. One prisoner was accommodated on the HSU due to the acute risk he posed to others.

Regarding diagnostic classification, 29% of those accommodated on the HSU were diagnosed with a major mental illness. 20% required short-term increased support for crisis intervention and were found not to have a mental illness. A further 10% were deemed to be feigning symptoms of mental illness in order to seek refuge on the HSU. 7% were diagnosed with a personality disorder and 4% of prisoners had a learning disability.

#### Outcome data: episodes of seclusion/isolation in SOCs

Table 2 shows the use of SOCs prior to and after the opening of the HSU. For the 11 months prior to the commencement of the HSU, the mean number of episodes of seclusion/isolation in SOCs per day was 2.2 (SD 1.0, 95% confidence interval 1.5 to 2.9); for the twelve months after the introduction of the HSU the mean number of episodes of use of SOC per day was 0.9 (SD 0.5, 95% confidence interval 0.6 to 1.2), ANOVA F = 14.9, df = 1, *p *< 0.001. This was a fall in mean episodes of use of SOCs per day by 59%. Because of the possible biasing role of 'outlier' months, non-parametric tests were used. The Mann-Whitney test yielded U = 7.0, Z = -3.485, *p *< 0.001 and the Kolmogorov-Smirnov test for extreme differences Z = 1.946, *p *= 0.001.

**Table 2:** Episodes of use of special observation cells (SOC) total and per day, and transfers from prison to hospital, before and after the opening of the high support unit (HSU)

Months	Number of episodes of use of SOC	Number of episodes of use of SOC per day	Transfers from prison to forensic hospital
	Before the opening of the HSU		

December 2009 -	-	-	0

January 2010	47	1.5	0

February 2010	84	3.0	0

March 2010	98	3.1	0

April 2010	131	4.3	2

May 2010	56	1.8	1

June 2010	65	2.2	1

July 2010	41	1.3	1

August 2010	58	1.9	0

September 2010	36	1.2	1

October 2010	42	1.4	1

November 2010	75	2.5	1

	After opening of the HSU		

December 2010	32	1.0	1

January 2011	36	1.2	0

February 2011	58	2.1	1

March 2011	35	1.1	1

April 2011	20	0.7	0

May 2011	15	0.5	0

June 2011	14	0.5	1

July 2011	31	1.0	0

August 2011	21	0.7	2

September 2011	38	1.3	2

October 2011	12	0.4	0

November 2011	28	0.9	0

#### Outcome data: transfers from the prison to the forensic psychiatric hospital

Table 2 shows the transfers from prison to the forensic hospital prior to and after the opening of the HSU. There were 8 transfers from the prison to the Central Mental Hospital in the 11 months prior to the commencement of the HSU and 8 in the 12 months after the HSU commenced. For the 11 months prior to the commencement of the HSU the mean number of transfers per month from the prison to the forensic hospital was 0.669 (SD 0.656) and for the 12 months after the introduction of the HSU the mean number of transfers was 0.672 (SD 0.779), ANOVA F = 0.0, df = 1, *p *= 0.991. The Mann-Whitney test yielded U = 71, Z = -0.061, *p *= 0.951 and the Kolmogorov-Smirnov test for extreme differences Z = 0.408, *p *= 0.996.

The mean length of stay in the CMH for those transferred from the prison in the eleven months prior to the commencement of the HSU was 71.8 (SD 69.4) days, and for the twelve months after the commencement of the HSU the mean length of stay was 47.0 (SD 32.7) days, ANOVA F = 0.8, df = 1, *p *= 0.377. The Mann-Whitney test yielded Z = -0.74, *p *= 0.46 and the Kolmogorov-Smirnov test for extreme differences Z = 0.50, *p *= 0.96.

After the introduction of the HSU, all transfers from the prison to the forensic hospital were from the HSU and 70% of transfers from the forensic hospital to the prison were to the HSU.

#### Main results

There has been a significant reduction in the frequency of use of SOCs in the prison. The mean daily or monthly rate of use of SOCs has fallen by 59% since the High Support Unit became operational.

There was no change in the rate of transfers from the prison to the forensic hospital demonstrating that the HSU was not used as a substitute for transfer to hospital where this was warranted. Because the pathway between prison and hospital was via the HSU, there was better communication and continuity of care, so that clinicians could have greater confidence in the physical and mental health and safety of patients returned from hospital to the prison.

#### Economic analysis

The initiative has been cost neutral to both the health service and prison service with no net change in total staff allocated to the prison by either service.

## Discussion

### Key results

The introduction of the HSU led to a significant fall in episodes of use of isolation cells (SOCs) across all of the prison. The care pathway for sentenced prisoners with severe mental illnesses from prison to the secure forensic psychiatric hospital also became more effective, with no increase or decrease in the rate of transfers but much improved communication and continuity because the HSU afforded better access to the in-reach mental health team. All transfers from the prison to the forensic hospital were from the HSU and the majority (70%) of discharges from the forensic hospital to the prison were back to the HSU, thereby ensuring better continuity of care and continuation of treatment programmes on return to the prison. We did not expect that transfers from prison to hospital would take place more quickly, though this proved difficult to measure. There was no acceleration of discharge back to the prison again in keeping with the role of the HSU as complimentary to hospitalisation, not a substitute for hospitalisation. This reorganisation and redeployment of manpower was cost neutral.

### Limitations

This is a description of an action research project to achieve change in a complex system. The measures of change are naturalistic observations. It was not possible to obtain data on incidents of self-harm or violence prior to the introduction of the high support unit, however the high support unit was not introduced to influence these problems, which are more likely to be reduced by screening on reception - as will be described in another study under way. Although the measures used here - number of episodes of use of SOCs and rates of transfers from prison to hospital are objective measures based on reliable registers, it is possible that many other factors may have mediated the changes observed. There were no changes in overall activity in the prison or in the forensic hospital over the period studied, nor were there any changes in legislation or service commissioning. The use of validated admission criteria [22,23] and triage criteria and procedures [24] minimised the risk that clinicians providing the in-reach service to the HSU had preferential access to the forensic hospital over clinicians providing in-reach clinics to other prisons without HSUs. It is possible that other, more subtle changes may have been relevant such as changes in the prevailing opinion formers and culture. These may or may not have been related to the action research approach.

There is an inherent risk that a HSU will be mistaken for a 'Hospital Wing' of a prison resulting in delayed transfer to hospital and accelerated discharge from hospital back to prison. The rates of transfer did not in fact change and length of stay did not fall significantly. There is also a risk that the unit will silt up with prisoners who are no longer in crisis but unwilling to return to ordinary location. Shortly after the setting up of the HSU, it became clear to the clinical team that a small minority of prisoners were feigning symptoms of mental illness so that they could be moved from the main prison in order to avoid threats from other prisoners. Many of these threats were related to outstanding drug related debts and were best dealt with by other means of protection. This was a benefit of the multi-agency mode of working.

Unfortunately, managing vulnerable and high risk prisoners in a dedicated facility carries an inevitable risk of adverse events occurring. The prison authorities need to be aware that a HSU is not just an area where prisoners are contained. It should be viewed as a functional and dynamic unit whose success will be influenced by increased relational security (staff to inmate ratios) in addition to improved environmental security. Regular inter agency meetings which share information and make joint decisions regarding admissions and discharges are an essential component in the optimal functioning of such a facility within a sentenced prison.

### Interpretation

The fall in the use of isolation cells across the prison can be attributed to greater confidence on the part of discipline staff that a safe alternative approach was available. It may also have been a side effect of the training provided - the minority of discipline officers under-going the training may have informally disseminated the knowledge and experience gained to their peers.

The improved effectiveness of the pathways for admission and discharge of sentenced prisoners between the prison and the forensic psychiatry hospital arose from the enhanced role of the forensic psychiatric in-reach team who were present almost daily in the prison while being based at the hospital and presenting cases for admission at the weekly referrals and admissions meeting at the hospital [24].

### Generalisability

A similar high support area had already existed for almost a decade in the main remand prison but the model had not been generalised to prisons for sentenced prisoners. The success of this project has led to positive comment from the Inspector of Prisons with a recommendation that similar units should be organised from within each of the other prisons.

Prison infirmaries and 'hospital wings' exist in various forms in other jurisdictions. These are seldom described in systems terms or audited for effectiveness, largely for want of an agreed performance indicator. The outcomes described here might constitute the basis for international benchmarking, if normalised for appropriate variables such as rate of committals to the prison and average length of stay.

## Conclusions

The participant action research model was an essential part of the success of this complex inter-agency change project. The agreed principles of risk-need responsivity, stratification of risk and need and the necessity of reducing the use of isolation cells (SOCs) emerged from the process as a common agenda and the High Support Unit, with co-operative working emerged as the common solution. Apart from managing vulnerable and mentally ill prisoners in a much more humanitarian environment, there is greater access to care and regular reviews by the prison in reach team from the National Forensic Psychiatry service and the Probation Service.

The introduction of the high support unit has achieved the goal of reducing the use of special observation cells (isolation cells) and has therefore improved compliance with human rights standards [5,6], as recommended [7,12,13]. The greater access to mental health services (Table 1) ensures equivalence of care for prisoners compared to the access they would have if in the community, while the pathways to and from hospital also emulate best practice in the community [15]. Prisons remain unsuitable places for people with severe mental illness. While much can be achieved by court liaison and diversion at the remand stage [23], once a severely mentally ill person has been sentenced the options available are limited and must focus on reducing the negative impact of the prison environment on mental health. For the future, the development of multi-agency aftercare services for vulnerable prisoners discharged to the community including housing and welfare with mental health services is a priority,

## Competing interests

The authors are all clinicians working in the services described.

## Authors' contributions

YG Design of the study. Establishing database. Assessment of cohort. Data collection. Analysis and interpretation of data. Drafting. AK Assessment of cohort. Data collection. EK Measurement of SOC use. HK Critical review of paper, design of quantitative analysis and data analysis. Drafting. DM Conception and design. Assessment of cohort. Drafting. All authors read and approved the final manuscript.
